# Controlling human platelet activation with calcium-binding nanoparticles

**DOI:** 10.1007/s12274-020-2912-8

**Published:** 2020-07-11

**Authors:** David Cabrera, Karen Walker, Sandhya Moise, Neil D. Telling, Alan G. S. Harper

**Affiliations:** 1 School of Pharmacy and Bioengineering, Keele University, Guy Hilton Research Centre, Thornburrow Drive, Hartshill, Stoke-on-Trent ST4 7QB, UK; 2 Central Electron Microscope Unit, School of Life Sciences, Keele University, Newcastle-under-Lyme, Staffordshire, ST5 5BG, UK; 3 Department of Chemical Engineering, University of Bath, Bath BA2 7AY, UK; 4 School of Medicine, Keele University, Newcastle-under-Lyme, Staffordshire, ST5 5BG, UK

**Keywords:** nanochelators, human platelets, open canalicular system, calcium signaling, nanoparticles

## Abstract

Human platelets aggregate at sites of blood vessel damage in response to a rise in their cytosolic calcium concentration. Controlling these cytosolic calcium rises would provide a method to inhibit platelet activation and prevent the unwanted blood clots that causes heart attack and strokes. Previously we have predicted that calcium accumulation within the lumen of an infolded portion of the platelet plasma membrane called the open canalicular system (OCS) is essential for maintaining this cytosolic calcium rise. Due to its nanometer dimensions of the OCS, it has been difficult to measure or interfere with the predicted luminal calcium accumulation. Here we utilise iron oxide magnetic nanoparticles coated with the known calcium chelator, citrate, to create calcium-binding nanoparticles. These were used to assess whether an OCS calcium store plays a role in controlling the dynamics of human platelet activation and aggregation. We demonstrate that citrate-coated nanoparticles are rapidly and selectively uptaken into the OCS of activated human platelets, where they act to buffer the accumulation of calcium there. Treatment with these calcium-binding nanoparticles reduced thrombin-evoked cytosolic calcium rises, and slowed platelet aggregation and clot retraction in human platelets. In contrast, nanoparticles that cannot bind calcium have no effect. This study demonstrates that the OCS acts as a key source of calcium for maintaining cytosolic calcium rises and accelerating platelet aggregation, and that calcium-binding nanoparticles targeted to the OCS could provide an anti-platelet therapy to treat patients at risk of suffering heart attacks or strokes.

## 1 Introduction

Human platelets are small, discoid, anucleate cells that orchestrate blood clotting at sites of blood vessel injury [1]. Clot formation is a multi-step process involving platelet adhesion to the damaged vessel wall, the recruitment of circulating platelets to form a platelet aggregate to block the hole in the vessel, activation of the blood coagulation to produce fibrin to strengthen the clot, and finally clot retraction to tightly seal the hole in the blood vessel wall [2]. Every one of these stages of platelet activation are triggered by rises in cytosolic calcium concentration ([Ca^2+^]_Cyt_). These calcium rises are triggered by thrombin and collagen (which are exposed in the wall of the damaged blood vessel) binding to receptors on the platelet surface. The activated receptors then initiate an intracellular signalling cascade that results in a rapid increase in the [Ca^2+^]_Cyt_ to trigger platelet activation and aggregation [3]. This central role for calcium in triggering the early events underlying blood clotting has led researchers to identify ways to inhibit cytosolic Ca^2+^ rises as a route to creating anti-platelet drugs that prevent platelet activation to protect against the unwanted blood clotting that occurs in patients suffering heart attacks and strokes [4].

One of the most striking features of human platelet ultrastructure is the open canalicular system (OCS), a system of invaginations of the plasma membrane that creates an elaborate series of tunnels through the platelet interior that are continuous with the extracellular fluid. However due to the complex geometry of the tunnel network, as well as its tiny lumen diameter (20–30 nm) [5], it has only been possible to study this structure by electron microscopy. This has led to the OCS being considered principally for its structural contributions to platelet activation, such as a site for granule fusion or as a membrane reservoir to allow shape change [6], membrane tether formation [7], or to facilitate platelet spreading over the subendothelial matrix [8]. Previously we showed that this structure may also play an important role in controlling platelet activation by acting as a dynamic Ca^2+^ store that can be recycled back into the platelet cytosol through Ca^2+^-permeable ion channels [9]. By potentiating and prolonging cytosolic Ca^2+^ signals this store might play a key role in regulating human platelet activation and aggregation [9]. We hypothesised that compounds that prevent Ca^2+^ storage within the OCS could slow platelet activation and therefore limit blood clotting. However due to the difficulty of assessing the chemical environment within the OCS in live platelets, a new experimental approach is required to examine whether Ca^2+^ rises within the OCS played a role in controlling human platelet activation and aggregation.

Iron oxide nanoparticles (IONPs) are bio-compatible magnetic particles that have been widely studied as potential theranostic agent. Due to their relatively simple synthesis [10], easily tunable physico-chemical properties [11], cheap cost of production [12] and minimal toxicity [13], a diverse range of IONP-based biomedical therapies and diagnosis approaches have been proposed in recent decades [14]. Owing to their nanometre size, IONPs are small enough to enter the OCS where they could be used to sense or alter the chemical environment. IONPs are commonly coated with citric acid to create a charged surface that allows them to be stable in polar solutions [15]. As citric acid is also an effective Ca^2+^-chelating agent that is commonly used in blood anti-coagulants [16], we hypothesized that citrate-coated IONPs could be utilized to create Ca^2+^ nanochelators that could enter the OCS lumen and buffer thrombin-evoked rises here. This would allow us to test the hypothesis that increases in Ca^2+^ concentration within the OCS are important in regulating human platelet activation.

In this paper, we produce calcium-binding IONPs and assess their ability to inhibit thrombin-evoked Ca^2+^ signalling and platelet activation. Through these experiments, we provide the first direct evidence that the OCS plays a key role in regulating Ca^2+^ signalling in human platelets, and that calcium-binding nanoparticles targeted to the OCS could be used to create novel anti-thrombotic drugs to prevent blood clotting in patients at risk of heart attacks and strokes.

## 2 Results and discussion

### 2.1 Characterisation of the physical properties of the IONPs

Prior to assessing their effect on human platelets, the physical and chemical properties of the IONPs used in this study were characterised with transmission electron microscopy (TEM) and dynamic light scattering-electrophoretic light scattering (DLS-ELS). These include citric acid-coated maghemite IONPs that were prepared in our labs (Cit/1-IO), as well as commercially-available citric acid-(Cit/2-IO) and amine-functionalised polyethylene glycol-coated (PEG-IO) IONPs. The PEG-coated IONPs were used to provide a comparison of the effects studied when the nanoscale dimensions were maintained, but the citric acid chelator was replaced with a non-chelating polymer surface coating.

**Figure 1:**
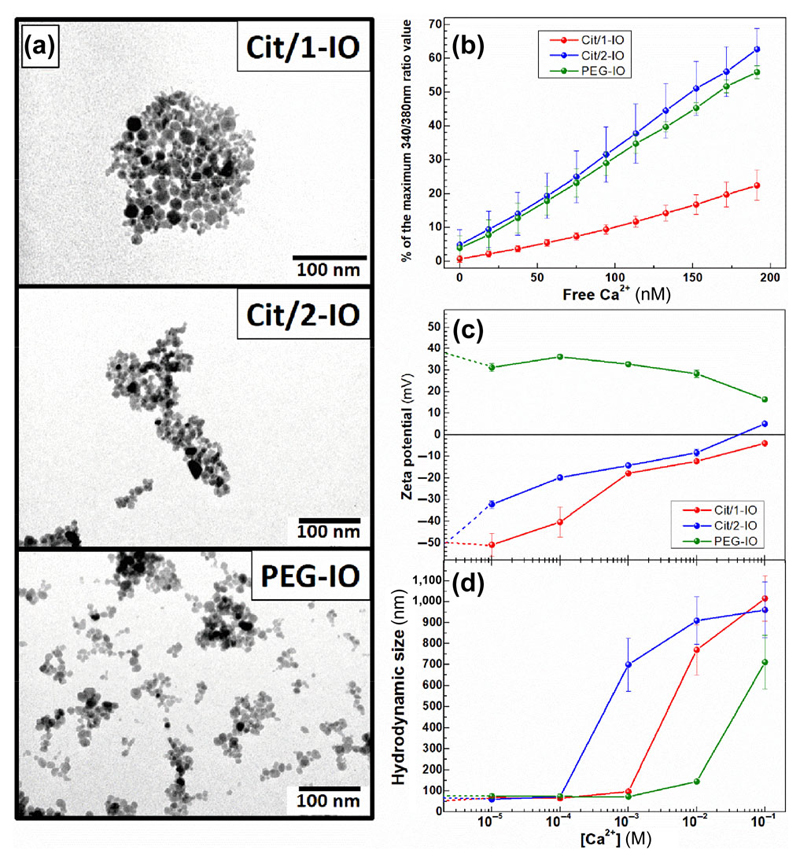
Characterisation of morphological and Ca^2+^-chelating properties of the IONPs. (a) Representative TEM images of Cit/1-IO, Cit/2-IO and PEG-IO nanoparticles. (b) Assessing the Ca^2+^ chelation properties of IONP samples. Equal concentrations of Cit/1-IO, Cit/2-IO and PEG-IO nanoparticles were added to a deionised water sample containing 250 μM EGTA and 3 μM Fura-2 pentapotassium salt held at 37 °C. Step-wise addition of known concentrations of CaCl_2_ were added to this EGTA-buffered solution and the free [Ca^2+^] was calculated using the MaxChelator program. Fura-2 fluorescence was measured at each extracellular [Ca^2+^] concentration, and normalised to % maximum of fluorescence measured in saturating Ca^2+^ concentrations. (c) Hydrodynamic size and (d) zeta potential measurements of the IONPs when suspended in solutions with different Ca^2+^ concentrations.

Figure 1(a) shows representative TEM images of the Cit/1-IO, Cit/2-IO and PEG-IO nanoparticles. All three nanoparticle sets showed a rounded morphology and had measured core sizes of less than 20 nm (Cit/1-IO = 15 ± 4 nm, Cit/2-IO = 9 ± 4 nm and PEG-IO = 10 ± 4 nm; (Fig. S1 in the Electronic Supplementary Material (ESM)). All of the IONP samples also exhibited comparable hydrodynamic sizes (*D*_H_) when dispersed in double-distilled water (ddH_2_0; 57, 62 and 75 nm for Cit/1-IO, Cit/2-IO and PEG-IO respectively; Fig. S1 in the ESM). As expected, the zeta potential (ζ) of these IONPs varies strongly depending on the IONPs coating, with citrate-coated IONPs being highly negatively charged (Cit/1-IO = –51.8 ± 0.4 mV and Cit/2-IO = –50.5 ± 0.2 mV), whereas the PEG-IO showed positive charge (+37.6 ± 0.5 mV). These values are in agreement with previously reported ζ values of particles coated with similar compounds and obtained from similar sources [17–19].

### 2.2 Characterisation of the Ca^2+^-binding capacity of IONPs

Citric acid is a widely-used anti-coagulant due to its Ca^2+^ chelating properties, however the ability of citrate-coated nanoparticles to effectively chelate Ca^2+^ has not been previously demonstrated. Experiments were performed to assess whether the citrate-coated IONPs were able to buffer Ca^2+^ levels in EGTA-buffered CaCl_2_ solutions containing the fluorescent Ca^2+^ indicator, Fura-2. IONP suspensions were added to the solution such that there was an equal concentration of iron (300 μM) in each sample, as measured by a ferrozine assay. Stepwise addition of known amounts of CaCl_2_ were added to the suspension, and the resultant increase in Fura-2 fluorescence was measured to assess the Ca^2+^ buffering abilities of the IONPs samples. The PEG-coated IONPs were used as a negative control to show the response from IONPs that do not bind Ca^2+^. As can be observed in Fig. 1(b), the commercially-sourced Cit/2-IO particles were not notably better at chelating Ca^2+^ in comparison to the PEG-coated particles, as there were similar increases in the Fura-2 fluorescence of the samples since the free [Ca^2+^] of the solution was increased. In contrast, the citrate-coated IONPs made in house were found to significantly reduce the increase in the Fura-2 fluorescence relative to its maximum value—suggesting that the citrate coating of this particle allows it to act as an effective Ca^2+^ chelator.

Further experiments were performed to examine the differential Ca^2+^ chelating capacity of the Cit/1-IO and Cit/2-IO nanoparticles. To assess if the different Ca^2+^-binding capacities were due to differences in the stability of these IONPs in solutions of different Ca^2+^ concentrations, measurements of ζ potential and hydrodynamic size (*D*_H_) were also performed when the IONPs were dispersed in solutions with a variety of Ca^2+^ concentrations. As can be seen in Fig. 1(c), the negative ζ potential of the Cit/2-IO particles rapidly decreased as the external Ca^2+^ concentration was increased into the micromolar range. In contrast, the Cit/1-IO maintained their ζ potential until the external Ca^2+^ concentration reached millimolar levels. Both Cit/1-IO and Cit/2-IO reached near zero ζ potentials at high millimolar levels suggesting that both particles have their negatively charged surfaces modulated by Ca^2+^, but requiring orders of magnitude different Ca^2+^ concentrations to do so. This difference in the reduction of the ζ potentials of the IONPs correlated with differences in the Ca^2+^ sensitivity of the *D*_H_ of the IONPs, with *D*_H_ of the particles starting to increase at lower Ca^2+^ concentrations in Cit/2-IO IONPs than Cit/1-IO (Fig. 1(d)).

In contrast, PEG-coated particles showed only minimal difference in ζ potentials and *D*_H_ as external Ca^2+^ concentration increased, suggesting this property is due to the differences in the surface coating of the IONPs and not the chemical composition of the IONP core. Our experiments show that the Cit/2-IO particles are prone to agglomeration when extracellular Ca^2+^ concentration is raised into the micromolar range, whilst the Cit/1-IO particles are stable in solution until millimolar Ca^2+^ concentrations. These data suggest that the greater Ca^2+^ sensitivity of the Cit/2-IO IONPs leads to their rapid aggregation when placed in Ca^2+^-containing solutions. This would lower the effective surface area of these citrate-coated particles available for Ca^2+^ binding. It appears that it is this inability to maintain an effective surface area in Ca^2+^-containing solutions that ultimately impairs their chelation capacity. The method of coating the magnetic nanoparticles with citric acid strongly determines the efficacy and stability of the grafting of this molecule to the nanoparticle surface. This will control the density, orientation and charge of citric acid coating on the nanoparticle surface, which could alter the nanoparticles calcium-binding capacity and stability in Ca^2+^-containing solutions. The Cit/1-IO nanoparticles were coated with citric acid using the optimised method described by Campelj et al. [2], and it is therefore possible that different coating methods used for the Cit/1-IO and the commercially-produced Cit/2-IO nanoparticles underly the discrepancy observed here through altering the effective surface density of citric acid on the surface of the nanoparticles. Alternatively citric acid may coat differently on the maghemite Cit/1-IO and magnetite Cit/2-IO nanoparticles. A comprehensive study of the conditions used to coat the nanoparticles with citric acid will be required to allow us to optimise the stability and calcium-binding capacity of the citrate-coated nanoparticles for use as an anti-platelet agent.

### 2.3 Activation-dependent incorporation of citrate-coated IONPs into the OCS of human platelets

TEM experiments were performed to assess if the IONPs could be incorporated into the OCS of human platelets. Platelet samples were prepared for TEM by incubating them for 10 min at 37 °C with the Cit/1-IO or Cit/2-IO nanoparticles. Samples were then either fixed directly, or stimulated for 1 min with thrombin and then fixed, to see if unstimulated and/or activated platelets could take up these IONPs into the OCS.

**Figure 2:**
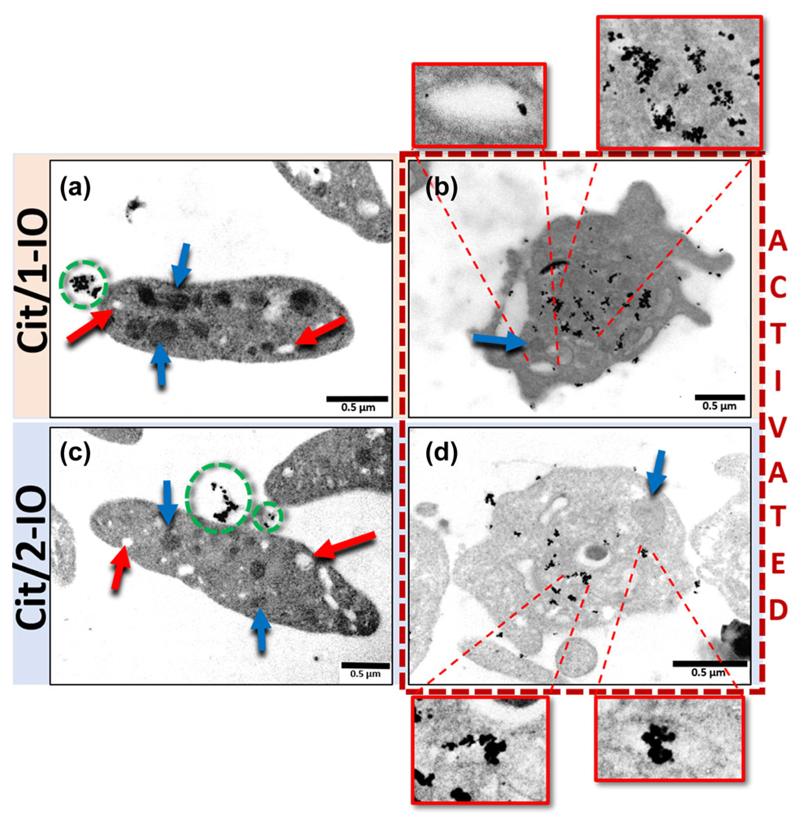
Citrate-coated IONPs are selectively and rapidly uptaken into the OCS of thrombin-stimulated platelets. Representative TEM images of platelets incubated with ((a) and (b)) Cit/1-IO and ((c) and (d)) Cit/2-IO nanoparticles at [Fe] = 300 μM at 37 °C for 10 min. Platelets in (a) and (c) were fixed whilst resting whereas in (b) and (d) were activated with 0.5 U/mL thrombin for 1 min before fixation. The OCS lumen are distinguishable from other subcellular regions as they appear to have a clear interior due to their continuity with the bulk extracellular fluid. Examples of OCS are shown by red arrows in the resting platelets and zoomed in red squares. Green dashed circles show IONPs outside the platelets. Blue arrows indicate granules.

Due to the lumen of the OCS having a diameter of around 20–30 nm in a resting platelet, they can only be observed through electron microscopy. In transmission electron microscopy images the OCS appears as irregularly-shaped, clear vacuoles occurring within the platelet interior, due to the inability of the extracellular fluid contained within the lumen to absorb or scatter electrons [5–8]. In contrast, the various platelet granules appear as electron dense structures of more regular size and shape. These structures are clearly observed in the unstimulated platelets as shown by red (OCS) and blue (Granules) arrows in Figs. 2(a) and 2(c). In the unstimulated platelets, it was observed that there was almost no uptake of any of the IONPs into the OCS, with any remaining IONPs after sample washing being found only in regions external to the platelet plasma membrane (Figs. 2(a) and 2(c)). This was consistent with the previous findings of Escolar et al. [8], who demonstrated that fibrinogen-coated gold nanoparticles do not permeate into the resting platelet.

In thrombin-activated platelets, the platelets begin to spread and lose their discord form and platelet granules are secreted, leading to these structures becoming less numerous inside the activate platelet (Figs. 2(b) and 2(d)). OCS channels also can become dilated as this structure reorganizes itself (Fig. S2 in the ESM). Thrombin-activated platelets were seen to have significant uptake of IONPs into the OCS. No nanoparticles were seen to be endocytosed into the remaining platelet granules (blue arrows; Figs. 2(b) and 2(d)). Instead both the Cit/1-IO and Cit/2-IO nanoparticles could predominantly be seen to densely accumulate within the lumen of the OCS—as seen by the low electron density and irregular shapes of the compartments (Magnified boxes; Figs. 2(b) and 2(d)). Additionally, closer examination of some of the electron microscope images showed particles in sections of the OCS connected via the pore region to the surface of the surface membrane (Figs. 2(a), 2(b) and 2(d); blue arrows). A small amount of Cit/1-IO and Cit/2-IO nanoparticles could also be observed to bind to the external face of the platelet plasma membrane (Figs. 2(b) and 2(d); Fig. S2 in the ESM shows additional images of particle-treated and untreated samples). This was also consistent with the previous findings of Escolar et al. [8]. This rapid loading is important to allow the coating of the IONPs to interact with the contents of the OCS during the immediate phase of platelet activation when cytosolic Ca^2+^ signalling elicits changes in platelet activation. The combination of sustained chelation efficiency and rapid uptake, make the Cit/1-IO IONPs suitable to be used as OCS-localised Ca^2+^ nanochelators in thrombin-stimulated human platelets. This result thus provides a method for testing our previously-stated hypothesis that the pericellular accumulation of Ca^2+^ occurs in the OCS, and that this is required for full platelet function.

There appeared to be no observable difference in the platelet uptake of the Cit/1-IO and Cit/2-IO particles, suggesting that the differences in the surface properties of these particles have no effect on their cellular uptake. Nanoparticle uptake into the OCS of human platelets will be determined by their ability to permeate through the pores of the OCS that link the invaginations of the OCS to the surface membrane of human platelets. This will be determined both by the number and size of the pores, as well as the negative surface charge of the platelet glycocalyx. Upon platelet activation, the pores of the OCS have been shown to widen upon stimulation with thrombin or Ca^2+^ ionophore [20, 21]. Additionally the number of pores connecting to the platelet surface has been shown to increase [22]. Both of these activation-dependent changes in the OCS pores could facilitate the easier permeation of our nanoparticles into this invaginated membrane system. Additionally, the surface negative charge created by glycosaminoglycans within the platelet glycocalyx would repel the negatively charged nanoparticles—this effect could be lessened upon activation by activation-dependent shedding of the glycocalyx [23] and shielding of the surface negative charge by Ca^2+^ binding to the glycocalyx [24]. Further work will be required to assess which of these factors most significantly affects platelet nanoparticle uptake, as this may provide valuable clues into how we can produce more effective OCS-targeted nanoparticles.

### 2.4 Treatment with the Cit/1-IO nanoparticles inhibits thrombin-evoked rises in cytosolic calcium levels in human platelets

Previous studies in our lab have demonstrated that thrombin-evoked platelet activation triggers a pericellular Ca^2+^ rise, which appeared to be localised within the OCS [9]. Inhibiting this pericellular Ca^2+^ rise by a number of methods has been consistently shown to inhibit thrombin-evoked increase in cytosolic calcium ([Ca^2+^]_cyt_) and thus interfere with platelet activation [4, 9]. Therefore, experiments were performed to assess whether preincubating human platelets with the Cit/1-IO nanoparticles could inhibit thrombin-evoked cytosolic Ca^2+^ signalling in a dose-dependent manner.

**Figure 3:**
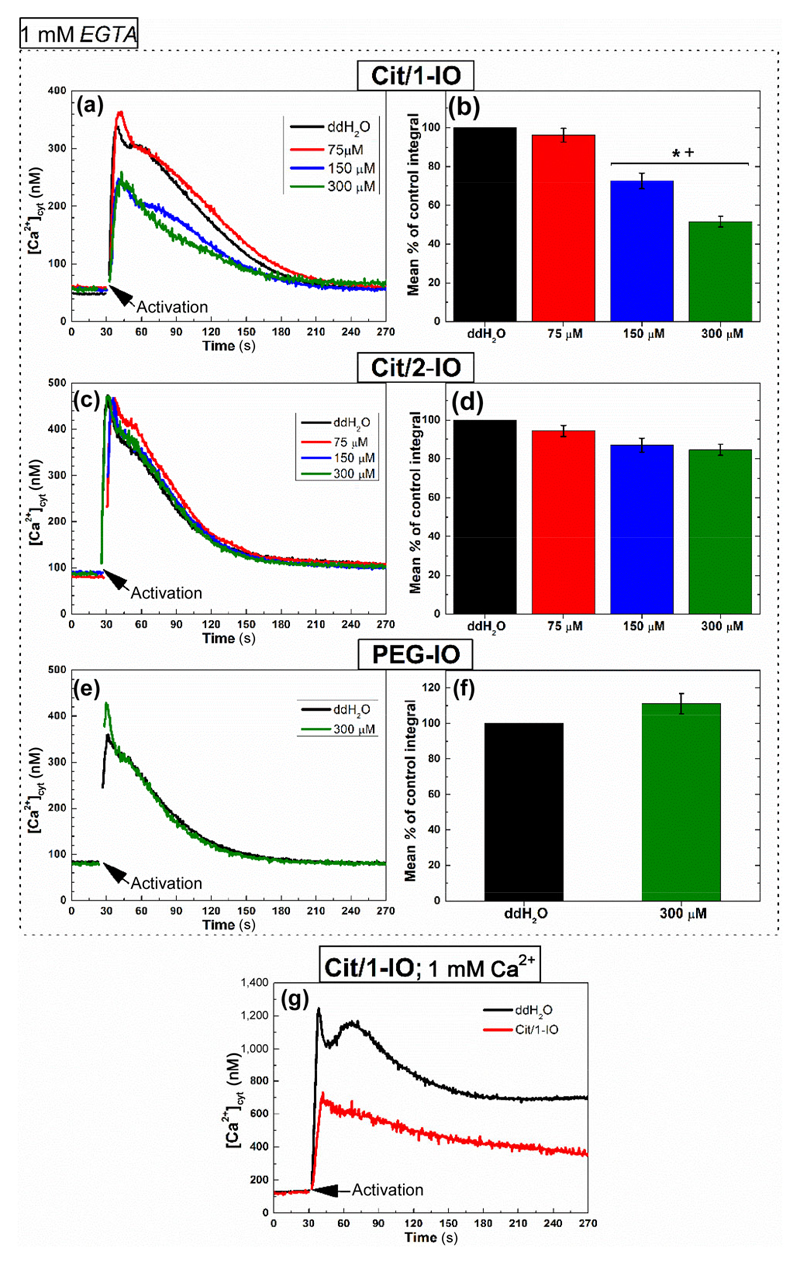
Thrombin-evoked rises in [Ca^2+^]_cyt_ are inhibited by preincubation with the Cit/1-IO Ca^2+^ nanoparticles, but not when incubated with the non-chelating Cit/2-IO or PEG-IO nanoparticles. Fura-2-loaded platelets were incubated with ((a) and (g)) Cit/1-IO, (c) Cit/2-IO, (e) PEG-IO nanoparticles at different concentration (75, 150 and 300 μM), or their vehicle, ddH_2_0, for 10 min at 37 °C. Extracellular Ca^2+^ was then either chelated by addition of 1 mM EGTA ((a)–(f)), or raised to 1 mM by addition of further CaCl_2_ (g). Platelets were then stimulated with 0.5 U/mL thrombin (black arrows). Column bar graphs display mean % of control of the integrals of the thrombin-evoked rises in [Ca^2+^] above basal when platelets were incubated with (b) Cit/1-IO, (d) Cit/2-IO, (f)PEG-IO nanoparticles. * *p* < 0.001 vs. control; + *p* < 0.05 between IONPs.

As can be seen in the representative traces shown in Fig. 3(a), preincubation of platelets with Cit/1-IO caused a dose-dependent inhibition of thrombin-evoked Ca^2+^ signals elicited in the absence of extracellular Ca^2+^, with the highest IONP doses (equivalent to the addition of 150 μM and 300 μM Fe) significantly inhibiting thrombin-evoked Ca^2+^ rises. As shown in Fig. 3(b), these higher IONP doses reduced the integral of the thrombin-evoked rise in [Ca^2+^]_cyt_ over basal to 73% ± 4% and 52% ± 3% of control, respectively (*n* = 7; *P* < 0.001, Fig. 3(b)—green and blue bars).

In contrast, preincubation of platelets with either the Cit/2-IO or PEG-IO nanoparticles elicited no significant effect on thrombin-evoked Ca^2+^ signals under the same condition, even at the highest particle concentrations used (Figs. 3(c)–3(f)). Control experiments in the absence of thrombin, also demonstrated that the Cit/1-IO nanoparticles did not directly activate platelets themselves. As shown in Fig. S3 in the ESM, addition of these IONP directly to platelets elicited no observable Ca^2+^ signal, demonstrating that the inhibitory effect observed previously was not due to IONP-induced activation of the platelets prior to thrombin addition. These data therefore suggest that it is not merely the presence of IONPs in the OCS that inhibits thrombin-evoked Ca^2+^ signalling, but rather the ability of the Cit/1-IO IONPs to chelate Ca^2+^ in this region. These results therefore demonstrate that the Cit/1-IO nanoparticles are able to elicit similar inhibitory effects on thrombin-evoked Ca^2+^ signalling to a range of other methods that have been utilised to prevent the pericellular Ca^2+^ accumulations [4, 9].

Further experiments assessed the ability of Cit/1-IO nanoparticles to inhibit thrombin-evoked cytosolic Ca^2+^ signals evoked in conditions of physiological extracellular Ca^2+^ concentration (1 mM). As can be seen in Fig. 3(g), preincubation with 300 μM Cit/1-IO nanoparticles significantly inhibited thrombin-evoked Ca^2+^ signals to 41.8% + 4.0% of control, respectively (*n* = 6; *P* < 0.05). These data therefore demonstrate that the Cit/1-IO Ca^2+^ nanoparticles can significantly inhibit human platelet cytosolic Ca^2+^ signalling when stimulated in physiological conditions. In contrast, the PEG-IO and Cit/2-IO nanoparticles were found to be ineffective and unable to be used as Ca^2+^-binding agents. Subsequent experiments focused on assessing whether the Cit/1-IO nanoparticles functioned through buffering Ca^2+^ rises within the OCS.

### 2.5 Cit/1-IO nanoparticles act as effective Ca^2+^ nanochelators as they buffer thrombin-evoked pericellular Ca^2+^ accumulations within the OCS

**Figure 4:**
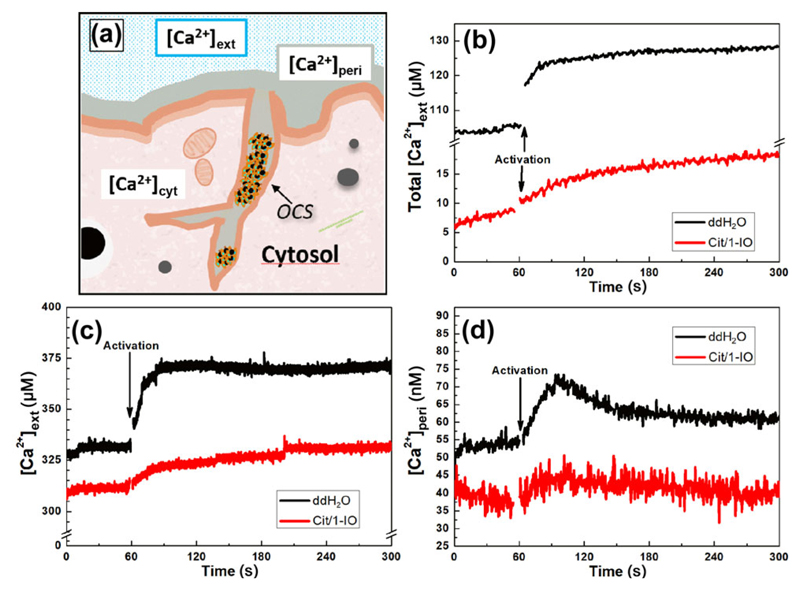
Cit/1-IO nanoparticles act as Ca^2+^ nanochelators by buffering thrombin-evoked rises in [Ca^2+^]_ext_ and [Ca^2+^]_peri_ concentration. (a) Schematic representation of the different cellular and extracellular compartments monitored by the fluorescent indicators. ((b)–(d)) Washed platelet suspensions containing either Fluo-4 salt (b) or Rhod-5N salt (c) were preincubated with either 300 μM Cit/1-IO or an equivalent volume of its vehicle, ddH_2_0, for 10 min at 37 °C. Extracellular Ca^2+^ was then either chelated by addition of 1 mM EGTA (b), or raised to 300 μM by addition of further CaCl_2_ (c). Platelets were then stimulated with 0.5 U/mL Thrombin (black arrows). (d) FFP-18-loaded human platelets were resuspended in supplemented HBS, and then were preincubated with either 300 μM Cit/1-IO or an equivalent volume of its vehicle, ddH_2_0, for 10 min at 37 °C. Extracellular Ca^2+^ was then chelated by addition of 1 mM EGTA and platelets were then stimulated with 0.5 U/mL thrombin (black arrows).

Experiments were performed to assess whether the Ca^2+^-chelating Cit/1-IO nanoparticles could successfully act as Ca^2+^ nanochelators by effectively buffering the thrombin-evoked pericellular Ca^2+^ accumulation in human platelets when loaded into the OCS. This was initially examined by assessing the effect of preincubation with these IONPs on thrombin-evoked rises in the extracellular Ca^2+^ concentration ([Ca^2+^]_ext_, Fig. 4). This was achieved by monitoring extracellular Ca^2+^ accumulation using the cell-impermeant salt form of the Ca^2+^ indicators, Fluo-4 and Rhod-5N. The different Ca^2+^ affinities of these indicators permit the assessment of Ca^2+^ removal in the extracellular fluid both in the absence of extracellular Ca^2+^ (Fluo-4) or in the presence of near-physiological levels of extracellular Ca^2+^ (300 μM; Rhod-5N).

As can be seen in Fig. 4, preincubation of human platelets with Cit/1-IO nanoparticles elicited a significant reduction in thrombin-evoked rises in [Ca^2+^]_ext_ both in the absence of extracellular Ca^2+^ (48.7% ± 5.6% of control; *n* = 5; *P* < 0.05; Fig. 4(b)), as well as in the presence of near-physiological Ca^2+^ levels (50.4% + 14.9% of control (*n* = 6; *P* < 0.05; Fig. 4(c)). These data demonstrated that the Cit/1-IO nanoparticles can significantly buffer thrombin-evoked rises in the Ca^2+^ contained within the extracellular fluid. However as both Fluo-4 and Rhod-5N are freely-diffusible through the extracellular fluid— they cannot distinguish Ca^2+^ located in the bulk extracellular fluid, from that contained within the lumen of the OCS or in the pericellular fluid surrounding the external face of the platelet plasma membrane. Therefore to assess pericellular Ca^2+^ rises more closely, experiments were performed using platelets in which the near-membrane fluorescent Ca^2+^ indicator, FFP-18, had been loaded into the extracellular face of the platelet plasma membrane using our previously described methodology [9]. As shown schematically in Fig. 4(a), this allows this fluorescent indicator to measure Ca^2+^ rises in the pericellular space surrounding the external face of the platelet plasma membrane and the contents of the OCS. Due to the high Ca^2+^ affinity of this dye, these experiments can only be performed in the absence of extracellular Ca^2+^ as it would become saturated at more physiological extracellular Ca^2+^ concentrations.

As shown in Fig. 4(d), preincubation of FFP-18-loaded platelets with Cit/1-IO IONPs elicited a significant decrease in thrombin-evoked rises in [Ca^2+^]_peri_, with the integral of this signal reduced to 27.1% + 7.9% of control (*n* = 5; *P* < 0.05). These data therefore indicate that the Cit/1-IO IONPs were able to effectively buffer thrombin-evoked rises in pericellular Ca^2+^ and thus are functional as effective Ca^2+^ nanochelators. Our previous single cell imaging of pericellular Ca^2+^ rises using Fluo-4 salt under the same conditions using the same methodology demonstrated that pericellular Ca^2+^ rises are not observed around the platelet surface membrane—but are instead found in an extracellular fluid compartment contained within the interior of the platelet, consistent with its presence in the OCS [4, 9]. These results indicate that the Cit/1-IO nanoparticles are able to buffer Ca^2+^ rises within the OCS. This is consistent with the TEM data in Fig. 2 that shows that Cit/1-IO is predominantly located inside the OCS. These results provide further evidence that the pericellular Ca^2+^ accumulation is localised inside the OCS, as we previously predicted [4, 9].

### 2.6 Treatment with the Cit/1-IO Ca^2+^ nanochelators inhibits platelet aggregation and clot retraction

**Figure 5:**
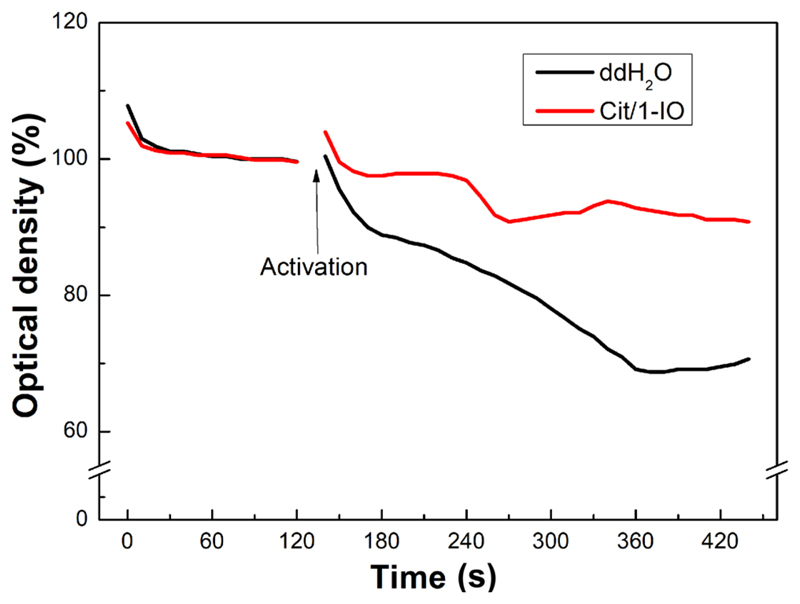
Treatment with the Cit/1-IO Ca^2+^ nanochelators inhibits platelet aggregation. Washed platelet suspensions resuspended in supplemented HBS were treated with Cit/1-IO nanoparticles such that the [Fe] of the samples was 300 μM, and incubated at 37 °C for 10 min prior to stimulation with thrombin (0.5 U/mL). These samples were compared against a vehicle-treated control in which the platelet suspension was incubated with an equal volume of ddH_2_O under the same conditions. Immediately prior to measurement, extracellular Ca^2+^ concentration was raised to 1 mM by addition of CaCl_2_. Platelet aggregation were then stimulated by addition of 0.5 U/mL thrombin (black arrow). This trace is representative of 5 independent experiments.

To assess the impact of the nanochelators on platelet functionality, experiments were performed to elucidate the effect of pre-incubation of human platelets with Cit/1-IO Ca^2+^ nanochelators on platelet aggregation and clot retraction. Conventional light transmission aggregometry utilises magnetic stirring to assess aggregation-induced light transmission changes through washed platelet and platelet-rich plasma samples stimulated with a platelet activator such as thrombin. However, due to the use of the magnetic IONPs, this was not a viable option in this case. Instead, experiments were performed utilising the alternative microplate-based aggregation assay [25]. This technique examines platelet aggregation by measuring changes in light absorbance of washed platelet samples stimulated with 0.5 U/mL thrombin. These studies demonstrated that Cit/1-IO slowed platelet aggregation to 51.0% ± 12.0% of control (*n* = 5; *P* < 0.05; Fig. 5), which is consistent with the degree of inhibition of Ca^2+^ signalling observed in the presence of 1 mM Ca^2+^ (Fig. 4(g)).

**Figure 6:**
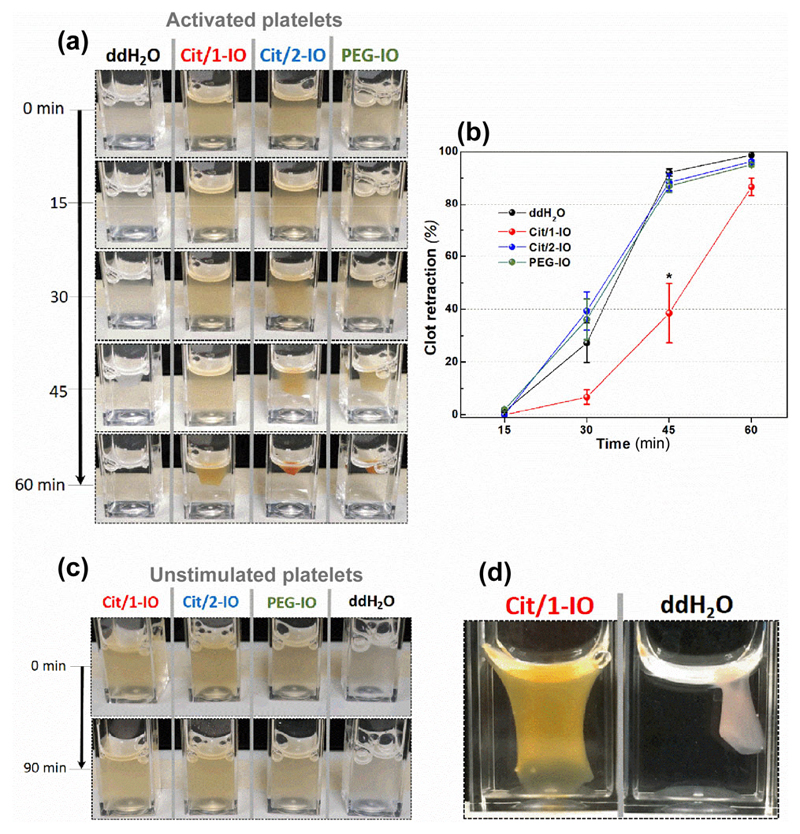
Treatment with the Cit/1-IO Ca^2+^ nanochelators slows thrombin-evoked clot retraction. Washed platelet suspensions resuspended in supplemented HBS were treated with Cit/1-IO, Cit/2-IO and PEG-IO nanoparticles such that the [Fe] of the samples was 300 μM, and incubated at 37 °C for 10 min prior to stimulation with thrombin (0.5 U/mL). These samples were compared against a vehicle-treated control in which the platelet suspension was incubated with an equal volume of ddH_2_O. (a) Representative clot retraction images from one experiment set and (b) mean values of clot retraction for each particle at 15, 30, 45 and 60 min. (c) Platelet exposure to IONPs in the absence of thrombin stimulation elicits no notable aggregation. (d) Representative clot retraction images from one experiment set of platelets in Ca^2+^ physiological conditions at 40 min after activation. (*n* = 4 for activated platelets, *n* = 3 for unstimulated platelets, *n* = 7 for platelets with fibrin in Ca^2+^ physiological conditions). *p < 0.05 vs. control.

Similarly, studies performed on washed platelet suspensions assessed the ability of the various IONP formulations to inhibit the retraction of the formed platelet aggregate. As shown in Fig. 6(a), IONPs disperse evenly through the platelet suspension prior to stimulation giving it a notable yellow colour. Prolonged incubation of the IONPs with the samples caused no notable activation of human platelets, with platelet suspensions remaining stable for the 90 min observation period (Fig. 6(c)). Upon thrombin addition, IONPs can be seen to become selectively cleared from the bulk solution and are instead found concentrated within the formed platelet aggregate, in agreement with the earlier TEM data confirming that IONPs are only taken up by activated platelets (Fig. 6(a)). This result shows that thrombin stimulation triggers uptake of all IONPs by platelets regardless of their surface chemistry. Despite IONP accumulation in all platelets, Cit/1-IO was the only nanoparticle sample that slowed the rate of the retraction of the platelet aggregate, with notable differences observed after 30 and 45 min of thrombin stimulation (Fig. 6(b)).

Further experiments were performed to assess the ability of Cit/1-IO nanoparticles to inhibit clot retraction when washed human platelet suspensions where suspended in HBS containing a physiologically-relevant extracellular Ca^2+^ concentration (1 mM) and 1 mg/mL fibrinogen. As shown in Fig. 6(d), aggregate retraction is significantly inhibited 40 min after thrombin stimulation (27.6% ± 8.1% of control, Fig. 5(d)). These data demonstrate that even in the presence of high extracellular Ca^2+^ concentrations found in plasma, the Cit/1-IO nanoparticles can still significantly inhibit platelet function through their ability to act as Ca^2+^ nanochelators within the OCS of human platelets.

In summary, these results demonstrate that the OCS lumen is a cellular compartment that plays an active role in controlling human platelet activation. Thus understanding how thrombin-evoked changes in the contents of the OCS lumen may be essential to understand the processes regulating *in vivo* thrombus formation. To achieve this, IONPs could be further functionalised with fluorescence indicators or reporter enzymes to act as novel sensors of the chemical environment within the OCS. Such particles would allow further investigations to assess how dynamic an environment the lumen of the OCS is upon platelet activation, and may provide additional routes to create OCS-targeted therapies. This finding also demonstrates that modifying the surface chemistry of magnetic nanoparticles to increase their calcium binding capacity would improve the biocompatibility of injected magnetic nanoparticles by preventing unwanted thrombotic reactions to these exogenous materials.

## 3 Conclusions

In this paper, we have demonstrated that enhancing the calcium-binding capacity of the surface of magnetic nanoparticles increases their potential to prevent the activation and aggregation of human platelets. The calcium-binding Cit/1-IO nanoparticles are able to inhibit platelet function due to their ability to rapidly enter the OCS upon human platelet activation, and act as Ca^2+^ nanochelators that buffer thrombin-evoked Ca^2+^ accumulations within the OCS. This has provided the first evidence that this cellular structure is an active regulator of platelet function through its ability to act as a source of Ca^2+^ that helps to maintain the cytosolic Ca^2+^ signals that elicit platelet aggregation and clot retraction. This ability of the Cit/1-IO nanoparticles to change the chemical environment within the OCS lumen has demonstrated that activation-dependent changes in the contents of the OCS lumen may play a key role in modulating human platelet function during thrombus formation. As little data is currently available on activation-induced changes in the composition of the OCS, further studies using nanoparticle to sense or alter changes in the chemical environment within the OCS may help identify additional roles for this understudied structure in regulating normal haemostatic reactions.

By acting as Ca^2+^ nanochelators, these nanoparticles are able to inhibit the rate of platelet aggregation and its subsequent retraction, providing evidence of the efficacy of an OCS-targeted anti-platelet therapy. A significant advantage of these nanoparticles is their simplicity, which would make them readily scalable for large-scale production. Additionally, being able to manipulate platelet activation by modifying the contents of the OCS lumen, would be particularly attractive as this structure is continuous with the blood plasma, thus preventing the need for cellular uptake. Therefore, this should make delivering therapeutic compounds into this region simpler than designing therapies that target intracellular proteins, which would need to permeate the cell membrane.

Further to this, the Ca^2+^ nanochelators could provide additional clinical functions beyond that offered by traditional anti-platelet therapies. Firstly, the use of a magnetic nanoparticle would allow their recovery using external magnetic gradients, in a technique known as blood magnetic filtration [26]. This could allow provision of an anti-platelet agent to limit further clotting in patients undergoing surgery to treat an acute cardiovascular event, as blood magnetic filtration would allow the effects to be easily reversed prior to percutaneous coronary intervention or bypass surgery. Additionally, due to the selective uptake of the Ca^2+^ nanochelators by activated platelets, these nanoparticles could also act as contrast agents to facilitate the imaging of blood clots [27], as well as the filtration of emboli from the peripheral circulation. Lastly these Ca^2+^ nanochelators could also improve thrombolytic therapies through use of magnetic hyperthermia to disrupt blood clots [28]. Thus citric acid coated nanoparticles provide a promising prototype for the development of new multi-functional clinical tools to diagnose and treat acute cardiovascular events.

## 4 Experimental

### 4.1 Materials

Fura-2/AM and FFP-18 K^+^ salt was from TEFlabs Inc. (Austin, TX). Thrombin was purchased from Merck Chemicals (Nottingham, UK). Fluo-5N/AM and K^+^ salts of Rhod-5N and Fluo-4 were purchased from Invitrogen (Paisley, UK). Apyrase was purchased from Sigma Aldrich (Gillingham, UK). All other reagents were of analytical grade. Commerical IONPs coated with citrate (referred to as Cit/2-IO) and PEG (referred to as PEG-IO) with amine terminal groups, respectively, were purchased at Chemicell-GmbH (Germany).

### 4.2 Custom citric acid-coated IONP

The IONPs denominated, Cit/1-IO, are maghemite nanometric particles purchased at Sigma-Aldrich as (iron(III) oxide, < 50 nm particle size). The nanoparticles were coated with citric acid using the protocol described elsewhere [29, 30].

### 4.3 Characterisation of IONPs core size

The core size of the IONPs was estimated by TEM using a JEOL JEM-1230 transmission electron microscope operated at 100 kV. A dispersion of each IONP sample were deposited upon a commercial carbon-coated carbon grid (Agar Scientific) and left to evaporate prior to imaging. 200 IONPs were randomly selected from each sample and measured using ImageJ software to determine their size. The brightness of the TEM images were corrected to standardise their backgrounds.

### 4.4 Iron quantification

The iron concentration of magnetic nanoparticles dispersions was determined using the Ferrozine method. IONPs were digested in 6 M nitric acid at 60 °C overnight. Afterwards, digested samples were incubated in 3.3 M hydroxylamine hydrochloride at room temperature for 2.5 h to reduce Fe^3+^ cations to Fe^2+^. Finally, samples were incubated in dark in a solution containing 1.75 mM ferrozin and 0.1 mM HEPES for 1 hour at room temperature, and their absorbance were measured in a plate reader at 562 nm. The absorbance was then compared with a calibration curve obtained from an iron solution standard.

### 4.5 Hydrodynamic size and zeta potential measurements

The hydrodynamic size (*D*_H_) of the IONP samples were measured by DLS in a Zetasizer Nano (Malvern) in ddH_2_O. A laser emitting red light at 633 nm acted as energy source, 173° was the angle between the sample and the detector. Measurements were performed in 1 mL disposable cuvette. Zeta potential was measured using the same instrument in a disposable capillary cuvette. Measurements were performed at an iron concentration of 0.02 mg/mL.

### 4.6 Ca^2+^ buffering capability of IONPs

300 μM of the IONPs were dispersed in 1.2 mL ddH_2_O containing 250 μM EGTA and 3 μM Fura-2 K^+^ salt and incubated in cuvettes for 10 min at 37 °C. The fluorescence intensity of the suspension was recorded at 37 °C using a Cairn Research Spectro-photometer (Cairn Research, Faversham, UK) using excitation wavelengths (*λ*_ex_) of 340 and 380 nm, and emission wavelengths (*λ*_em_) of 515 nm. Variations in 340/380 nm ratio were monitored upon the stepwise addition of CaCl_2_ to the dispersion and normalised to the saturation 340/380 nm ratio value for each sample. The free [Ca^2+^] concentration of the buffered solution was calculated using MaxChelator software (Standford University, USA).

### 4.7 Preparation of washed human platelet suspensions

This study was approved by the Keele University Research Ethics Committee. Blood was donated by healthy volunteers who had provided written informed consent. Blood was collected by venepuncture and mixed with one-sixth volume of acid citrate dextrose (ACD) anticoagulant (85 mM sodium citrate, 78 mM citric acid, and 111 mM D-glucose). Platelet-rich plasma (PRP) and washed platelets were prepared according to previously-published protocols [9]. PRP was prepared by centrifugation of anticoagulated blood for 8 min at 1,500*g*, to which 100 μM Aspirin and 0.1 U/mL apyrase were then added. Washed platelet suspensions were obtained by centrifugation of PRP at 350*g* for 20 min, and the platelet pellet was then resuspended in a HEPES-buffered saline (HBS; pH 7.4, 145 mM NaCl, 10 mM HEPES, 10 mM d-glucose, 5 mM KCl, and 1 mM MgSO4) supplemented on the day with 1 mg/mL bovine serum albumin, 10 mM glucose, 0.1 U/mL apyrase, and 200 μM CaCl_2_ (Sup-HBS).

### 4.8 Subcellular localisation of platelet-associated IONPs

Platelets suspended in sup-HBS were incubated with each two citric acid coated IONPs (Cit/1-IO and Cit/2-IO, [Fe] = 300 μM) at 37 °C for 10 min. Immediately after, a part of platelets suspensions were treated with of EGTA (1 mM) and stimulated with thrombin (0.5 U/mL) in analogous manner to [Ca^2+^]_cyt_ measurements. 1 min after the activation, all platelet samples were fixed following the protocol described by White et al. [8].

Briefly, platelet suspensions were mixed with equal volume of White’s buffer containing 0.01% of EM grade glutaraldehyde (GTA) (v/v) and incubated for 15 min at 37 °C. Immediately after, this pre-fixation buffer was substituted by centrifugation for White’s buffer containing 2.5% GTA (v/v) and platelet suspensions were kept at 4 °C for 1 h. Following the fixation, samples were washed 3 times in 0.1 mM sodium cacodylate buffer containing 2 mM calcium chloride (pH 7.4). In between each wash the samples were centrifuged before removing the excess buffer and then resuspended in the newly added buffer. The samples were then post fixed in 0.1% OsO_4_ in the same buffer for 1 hour at room temperature before being washed again 3 times with the same buffer using the same method as previously. Samples were subsequently suspended in 3% agar (w/v) aqueous solution to provide a suitable media to support the samples through further processing. The samples in agar were then dehydrated through a graded ethanol series of increasing concentration (70%, 80%, 90%, 100% and Dry 100%) for 15 min in each concentration. Finally, the samples were infiltrated through increasing concentrations of ethanol to SPURR resin before having 3 changes of pure SPURR resin and being embedded in moulds with fresh prepared SPURR resin, and then polymerised at 60 ° C for 16 h. Ultrathin section of 70 nm thickness were then taken using a Leica Ultracut UCT ultramicrotome onto 200 mesh thin bar copper grids using a diamond knife. The sections were examined using a JEOL JEM-1230 transmission electron microscope operated at 100 kV. A digital Software Imaging System (SIS) Megaview III camera was used to capture electron micrographs with SIS iTEM software. Brightness of the TEM images was corrected to standardize their background.

### 4.9 Monitoring of thrombin-evoked rises in extracellular Ca^2+^ concentration

The release of Ca^2+^ to the extracellular medium in the absence of extracellular Ca^2+^ was monitored in washed platelet suspensions resuspended in a sup-HBS containing 1 mM EGTA, by addition of 2.5 μM Fluo-4 K^+^ salt to the suspension immediately prior to the start of experiments. Fluorescence were recorded as for Fura-2 above but with *λ*_ex_ = 485 nm and *λ*_em_ between 515 and 565 nm. The release of Ca^2+^ to the extracellular medium in the presence of 300 μmol/L extracellular Ca^2+^ was monitored by addition of 2.5 μmol/L Rhod-5N to washed platelet suspensions immediately prior to the start of experiments. Fluorescence was monitored as for Fura-2 above but with excitation at 550 nm and collecting emitted light of wavelengths between 570 and 640 nm. Fluo-4 and Rhod-5N data were calibrated to measure the total Ca^2+^ concentration (buffered and free Ca^2+^) in the extracellular medium as previously described [3].

### 4.10 Monitoring of thrombin-evoked rises in pericellular Ca^2+^ concentration

Thrombin-evoked changes in [Ca^2+^]_peri_ was monitored in platelets loaded with FFP-18 salt using a method to specifically label extracellular membrane [9]. Sup-HBS platelets suspensions were treated with FFP-18 (5 μM) for 5 min at room temperature. Later, platelets were split in 1 mL aliquots to which 250 μL of ACD was added. Platelets were centrifuged at 8,000*g* for 30 s and supernatant was removed and the pelleted cells were resuspended in sup-HBS. Fluorescence was recorded as for Fura-2 above. Changes in [Ca^2+^]_peri_ were monitored using the 340/380 nm fluorescence ratio.

### 4.11 Monitoring thrombin-evoked changes in cytosolic Ca^2+^ concentration

Thrombin-evoked changes in cytosolic Ca^2+^ concentration ([Ca^2+^]_cyt_) were estimated using Fura-2-loaed human platelets. PRP was incubated with Fura-2/AM (2 μM) for 45 min at 37 °C. PRP was then centrifuged at 350*g* for 20 min, and the platelets were then re-suspended in sup-HBS. Fluorescence was recorded from 1.2 mL stirred aliquots of platelet suspension at 37 °C with a Cairn Research Spectrophotometer (Cairn Research, Faversham, UK) using *λ*_ex_ of 340 and 380 nm, and *λ*_em_ of 515 nm. Changes in [Ca^2+^]_cyt_ were calibrated using the methodology of Grynkiewicz et al. [31].

### 4.12 Quantification of thrombin-evoked rises in [Ca^2+^]_Cyt_, [Ca^2+^]_Peri_ and [Ca^2+^]_Ext_

Quantification of thrombin-evoked rises in [Ca^2+^]_cyt_, [Ca^2+^]_peri_ and [Ca^2+^]_ext_ were quantified by integration of the change in fluorescence records above basal fluorescence with respect to time for 3 min after thrombin addition. Basal fluorescence was defined as the mean [Ca^2+^] recorded for 10 s prior to thrombin addition (as shown schematically in Fig. S4 in the ESM).

### 4.13 Platelet aggregometry

Washed human platelet suspension resuspended in supplemented HBS were preincubated with either 300 μM Cit/1-IO nanoparticles or an equivalent volume of ddH_2_0. The external Ca^2+^ concentration was then raised to 1 mM. 100 μL aliquots were then dispensed into 96-well plates. Light absorbance was measured on BioTek Synergy microplate reader set up to keep plates at 37 °C. Basal absorbance was measured every 10 s for 5 min to allow for measurement of maximum absorbance of both samples (*A*_max_). Platelets were then stimulated by addition of 0.5 U/mL thrombin and light absorbance read every 10 s for a further 10 min. A minimum absorbance (*A*_min_) was measured simultaneously from a platelet-free supplemented HBS sample to which 300 μM Cit/1-IO nanoparticles or an equivalent volume of ddH_2_0 had been added. Data were normalised to % optical density using the following formula

(1) $$\%\;\text{Optical density = 100}\cdot \frac{A-A_{\min}}{A_{\max}-A_{\min}}$$

The maximum decrease in optical density obtained during the experiment was compared between samples.

### 4.14 Clot retraction measurements

1.2 mL of washed platelets suspensions resuspended in supplemented HBS were aliquoted into cuvettes and incubated with the different IONPs ([Fe] = 300 μM) at 37 °C for 10 min. Platelets were then stimulated by the addition of 0.5 U/mL thrombin and clot formation and retraction was then monitored taking photos every 15 min for 90 min at room temperature. Images were later analysed with ImageJ by calculation of the relative area occupied by the clot in the front face of cuvettes.

### 4.15 Statistical analysis

Blood from two to five donors were used in each experiment. *n* signifies independently tested platelet samples. Values are reported as mean ± SEM, except for IONPs physicochemical and morphological characterisation, which are mean ± SD. Statistical significance was assessed by one-way ANOVA with a *post hoc* Tukey test of repeated measurements. A *P* < 0.05 was considered significant.

## Supplementary Material

Supplementary material
