# Supplementary material for: Controlling human platelet activation with calcium-binding nanoparticles
Source: Nano Res. Author manuscript; Available in PMC 2021 Jan 19. (PMC7116604; doi:10.1007/s12274-020-2912-8)
Supplement: Supplementary material [file EMS109143-supplement-Supplementary_material.pdf]

Electronic Supplementary Material

Controlling human platelet activation with calcium-binding nanoparticles

David Cabrera[1], Karen Walker[2], Sandhya Moise[1,3], Neil D. Telling[1], and Alan G. S. Harper[1,4] (✉)

[1] *School of Pharmacy and Bioengineering, Keele University, Guy Hilton Research Centre, Thornburrow Drive, Hartshill, Stoke-on-Trent ST4 7QB, UK*
[2] *Central Electron Microscope Unit, School of Life Sciences, Keele University, Newcastle-under-Lyme, Staffordshire, ST5 5BG, UK*
[3] *Department of Chemical Engineering, University of Bath, Bath BA2 7AY, UK*
[4] *School of Medicine, Keele University, Newcastle-under-Lyme, Staffordshire, ST5 5BG, UK*

Supporting information to https://doi.org/10.1007/s12274-020-2912-8

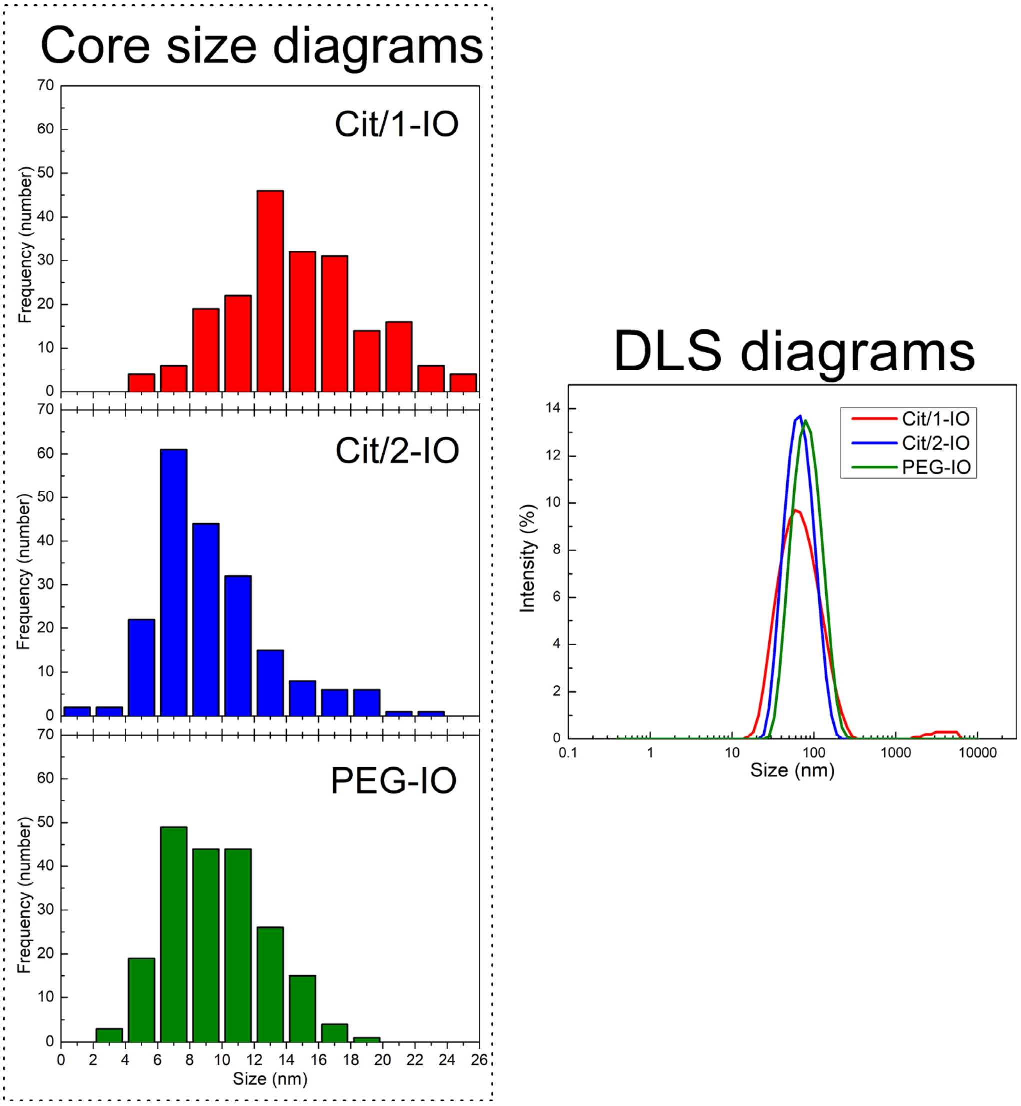

Figure S1 Core size and hydrodynamic size diagrams of Cit/1-IO, Cit/2-IO and PEG-IO nanoparticles.

Address correspondence to a.g.s.harper@keele.ac.uk

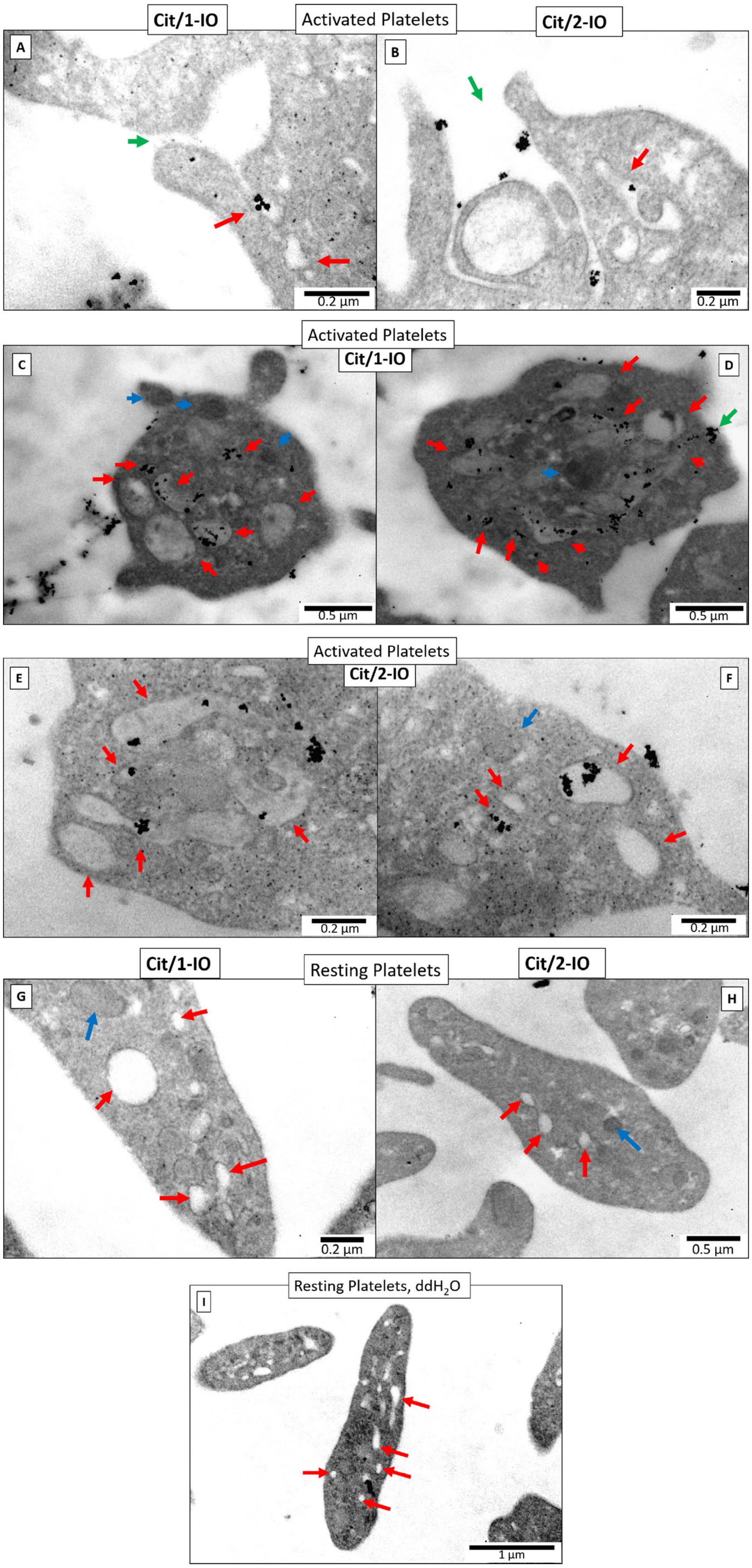

Figure S2 High-magnification TEM images of citrate-coated IONPs in resting and activated platelets. IONPs are observed in the OCS of thrombin-stimulated, but not resting platelet samples. A resting untreated cell image is shown for comparison. Note: to allow better visualization, the images for this figure are set out across multiple pages. Red arrows: Open Canalicular System. Blue arrows: granules. Green arrows: nanoparticles entering the OCS. Images obtained from three different donors. (multiple page figure)

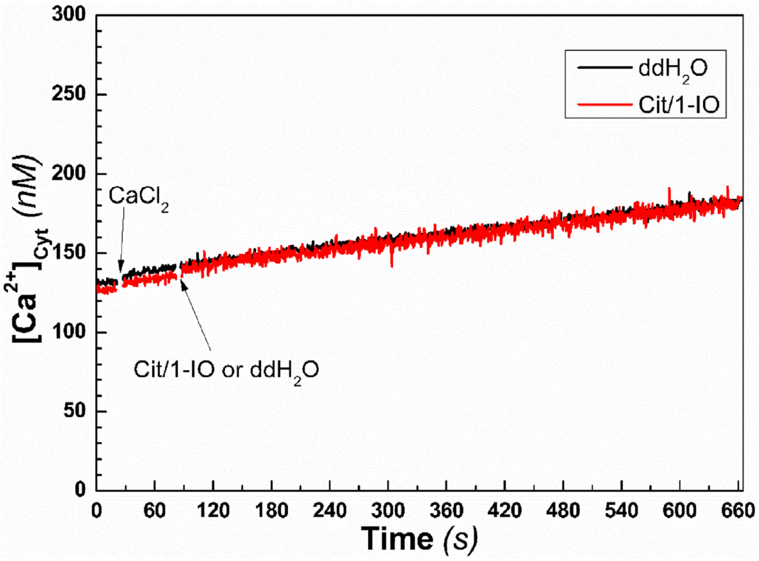

Figure S3 Representative measurement of $[Ca^{2+}]_{Cyt}$ levels of resting platelets exposed to Cit/1-IO using Fura-2AM. To offer a more disadvantageous scenario, Ca^{2+} concentration of platelet sup-HBS suspensions was top up to 1 mM Ca^{2+} by addition of $CaCl_2$. Afterwards, Cit/1-IO nanoparticles ([Fe] = 300 μM) were added into the 1.2 mL platelets aliquots and $[Ca^{2+}]_{Cyt}$ were monitored for 10 min. $S[Ca^{2+}]_{Cyt}$ of Cit/1-IO treated platelet were insignificantly different against control. (n =5; P > 0.05)

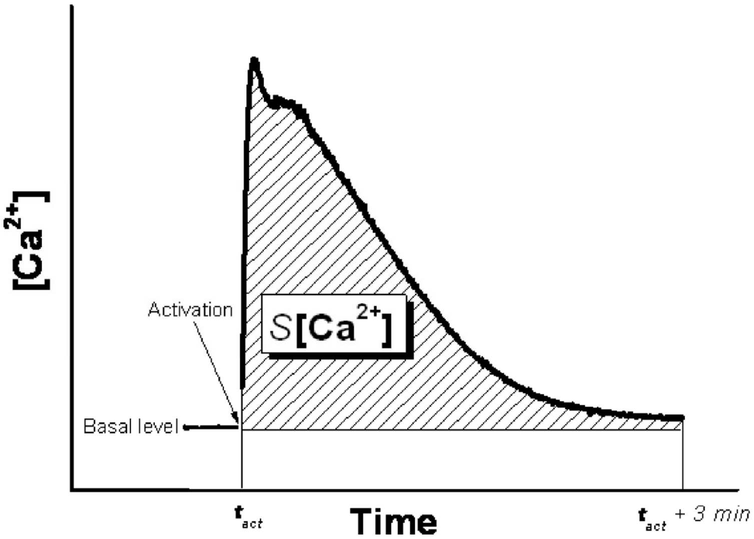

Figure S4 Graphical guide of the applied method to quantify thrombin-evoked $[Ca^{2+}]$ rises in different regions of the platelet dispersions. Dashed area represents the surface below the curve considered for $[Ca^{2+}]$ rises quantification.
